# Supplementary material for: Efficacy of Penicillin–Streptomycin Brands against Staphylococcus aureus: Concordance between Veterinary Clinicians’ Perception and the Realities
Source: Antibiotics (Basel). 2023 Mar 14;12(3):570. doi: 10.3390/antibiotics12030570 (PMC10044686; doi:10.3390/antibiotics12030570)
Supplement: Supplementary file 1 [file antibiotics-12-00570-s001.zip › antibiotics-2259276-supplementary.pdf]

Supplementary file S1. The questionnaire used to assess veterinarians' perceptions, practices, and knowledge of antibiotic quality, use and brand prescribing at Bishoftu and Sebeta, Central Ethiopia

**Question**		**Participants response (5-point Likert scale)**				
		Strongly agree	Agree	Neutral	Disagree	Strongly Disagree
**Perception of veterinary antibiotics quality and brand prescribing**						
Agree that some antibiotics are of poor quality in the market						
Antibiotics from western countries perceived as better quality than eastern countries						
Penstrep imported from the western country (E.g., UK) perceived as better quality than the Eastern countries (E.g., China)						
Generic antibiotics perceived as equivalent quality to branded antibiotics						
Generic antibiotics perceived as substandard drugs						
Prescribe antibiotics by international nonproprietary name						
Prescribe antibiotics by brand name						
Prescribe brands of penstrep						
Variation in clinical improvements among brands of penstrep						
Which brands of penstrep showed better clinical improvements?	Pen & Strep (Norbrook)					
	Penstrep (Chengdu Quiankun)					
	Pro & Strep (Hebei Yuanzheng)					
Which brands of penstrep mostly prescribed?	Pen & Strep (Norbrook)					
	Penstrep (Chengdu Quiankun)					
	Pro & Strep (Hebei Yuanzheng)					
**Knowledge and perception of antibiotics and their use**						
Penstrep indicated for both Gram-positive and Gram-negative bacterial infections						
Oxytetracycline indicated for gastro-intestinal and respiratory bacterial infections						
Sulfa drugs indicated for diarrheic cases						
Antibiotics indicated for prophylactic use for severe viral cases						
**Antibiotic prescribing Practices**						
Perception of antibiotic overuse in the dairy farms						
Perception of antibiotic overuse in the veterinary clinics						
